# Factors associated with age of diagnosis of autism spectrum disorder among children in Saudi Arabia: new insights from a cross-sectional study

**DOI:** 10.1186/s13104-022-06035-x

**Published:** 2022-05-10

**Authors:** Fahad M. Alnemary, Faisal M. Alnemary, Gabriela Simon-Cereijido, Hesham M. Aldhalaan, Anthony Hernandez, Ahmed Alyahya, Shuliweeh Alenezi

**Affiliations:** 1 Department of Special Education, Taif University, Taif, Saudi Arabia; 2 Autism Center of Excellence, Riyadh, Saudi Arabia; 3 Department of Communication Disorders, College of Health & Human Services, California State University, Los Angeles, USA; 4 Center for Autism Research, King Faisal Hospital and Research Center, Riyadh, Saudi Arabia; 5 Department of Educational Foundation & Interdiv Studies, Charter College of Education, California State University, Los Angeles, USA; 6 Department of Psychiatry, College of Medicine, King Saud University, Riyadh, Saudi Arabia; 7 SABIC Psychological Health Research and Applications Chair (SPHRAC), Department of Psychiatry, College of Medicine, King Saud University, Riyadh, Saudi Arabia

**Keywords:** Autism, Age of diagnosis, Services, Saudi Arabia

## Abstract

**Objectives:**

Research examining the age of diagnosis of autism spectrum disorder (ASD) and its influencing factors mostly originate from developed Western countries, providing little to no systematic information about the understanding and management of ASD in the rest of the world. The present exploratory study examined the influence of child and family characteristics on the age of ASD diagnosis in Saudi Arabia.

**Results:**

The median age at diagnosis was 3.0 years and was associated with some child and family characteristics. A 1 year increase in child’s age was associated with a 0.1 year increase in age of diagnosis (95% CI 0.05, 0.12). Children who did not respond to their name were diagnosed 0.3 years earlier than other children (95% CI − 0.60, − 0.05), and engaging in challenging behavior was associated with a 0.5 year increase in age of diagnosis (95% CI 0.20, 0.81). A lack of comorbidity was associated with a 0.6 year increase in the age of diagnosis compared to the diagnosis age of children with comorbidity (95% CI 0.13, 1.01). Finally, those residing outside of Saudi Arabia were diagnosed with ASD 0.9 years earlier than those residing in Saudi Arabia (95% CI − 0.171, − 0.11).

**Supplementary Information:**

The online version contains supplementary material available at 10.1186/s13104-022-06035-x.

## Introduction

Autism spectrum disorder (ASD) is a complex neurodevelopmental disorder characterized by wide-ranging deficits in social communication and social interactions as well as restricted, repetitive patterns of behavior, interests, or activities. ASD occurs in one of every 160 children, with 4:1 male:female ratio, worldwide and has a significant impact on the affected individual, his or her family, and society [1–3]. Previous findings have suggested that early intervention could substantially minimize the impact of ASD [2, 4]. Therefore, accessing early intervention services is warranted after early diagnoses of ASD [5]. However, despite advancements in early developmental screening and comprehensive diagnostic evaluations, many children are not diagnosed until later in life [6]. Identifying the factors associated with the age of ASD diagnosis is thus essential to understanding the barriers to accessing diagnostic and intervention services.

Research examining the age of ASD diagnosis and the factors affecting it originates primarily from developed Western countries [7], providing little to no systematic information about the understanding and management of ASD in the rest of the world, particularly Saudi Arabia. Hussein et al. (2011) compared 20 children with ASD in Saudi Arabia with 28 children in Egypt and found that the age at which parents started being concerned about their child's development was younger for Saudi children; however, Saudi children were diagnosed later than their Egyptian counterparts [8]. More recently, Murshid (2011) recruited 324 families of children with ASD from several major cities, namely, Riyadh, Jeddah, and Dammam, and found that most children were diagnosed before the age of 5 years (78%). The percentages of children who were diagnosed before 5 years old were similar across cities [9]. These studies by Hussein et al. (2011) and Murshid (2011) shed light on some aspects of ASD diagnosis in Saudi Arabia; however, the ability to draw inferences from these studies is limited.

This exploratory study sought to address this gap in the literature to provide recommendations to enhance current and prospective ASD services and research in Saudi Arabia. We examined parents’ experiences with the process of their children’s ASD diagnosis. We aimed to determine which child and family factors were associated with the age of ASD diagnosis.

## Main text

### Materals and methods

#### Study design and sampling

This is a cross-sectional study; an online survey was used to collect data between April and June 2014 from parents of children who had ASD and were younger than 18 years. A Google search was conducted to identify support groups for parents of children with autism. The search terms were “autism,” “parent support groups,” and “Saudi Arabia.” Relevant websites and social networking sites were visited to determine the e-mail address of the contact person(s). A total of six parent support groups shared the survey link through their social networking sites, which was completed by 238 parents. Our aim in this study is to explore the ASD services in Saudi Arabia. Therefore, 33 surveys were excluded because the responding parents were non-Saudi residents living in a different country. This study was approved by the institutional review board (IRB) of Taif University, Taif, Saudi Arabia, and the IRB of the University of California, Los Angeles study and the use of these data.

#### Survey development

The full survey was based on anecdotal reports from clinical experts and families and the current literature. The survey included the following two sections.

##### Family

The first section included family-related questions such as parents’ age and educational level and the family’s geographic location. The family's location was categorized into one of three groups: a major city, non-major city, or a location outside of Saudi Arabia. The major cities had the largest populations, including Riyadh, Jeddah, and Dammam. Other cities were considered non-major cities [10].

##### Child

The second section included questions about the child such as age, gender, presence of ASD red flags (e.g., not responding to name, making no/poor eye contact, and exhibiting language regression), primary diagnosis, comorbid conditions (e.g., intellectual disability, attention-deficit/hyperactivity disorder), age of ASD diagnosis, city of diagnosis, cost of diagnosis, and the severity of symptoms. The Parental Concerns Questionnaire (PCQ) was used to assess the severity of symptoms [11]. The PCQ consists of 13 items and uses a four-point scale (1 = no problem, 2 = mild, 3 = moderate, 4 = severe problem) with a total ranging from 13 (mild symptoms) to 52 (severe symptoms) to measure the extent to which each of the core and behavioral symptoms of ASD has been a problem for the child. Despite the adequate psychometric properties of the scale, a factor analysis of the PCQ was conducted in the present study. The maximum likelihood extraction method was utilized because the data were relatively normally distributed [12], while a scree plot excluding the inflexion point’s criterion was used to determine the number of factors [13]. The 13-item scale appeared to underlie one factor, with a reliability above the acceptable level (Cronbach’s alpha = 0.80). Three individuals independently translated the survey into Arabic using forward translation [14]. They worked individually to translate the survey and then met to discuss the translations and create one Arabic version.

#### Pilot testing

After developing the initial survey, we conducted a small pilot testing to examine the readability of the questionnaire. A total of six parents of children with ASD, aged 4–18 years, participated in this pilot study. After completing the initial survey, each parent met with the first author (F.M.A) to review and comment on the content and readability. All feedback was considered when finalizing the survey (Additional file 1).

#### Analysis

Frequencies, means, and standard deviations, and medians with ranges were calculated for all demographic and clinical variables as appropriate. Kruskal–Wallis test was used to determine whether the age at diagnosis varied significantly based on the variables of interest. Linear regression analysis was performed to characterize the relationship between each demographic and clinical predictor and age at ASD diagnosis. Each predictor was entered into a univariate model with the outcome variable to calculate the unadjusted regression coefficients and associated 95% confidence intervals (CIs). The predictors that were found to be significant at an alpha level = 0.05 were included together in a multivariate model. The multivariate model was developed through backward elimination to identify which combination of predictors best explained age at ASD diagnosis. All variables were initially included in the model, but those that caused the smallest reduction in R^2^ were subsequently removed. This process continued until there were no variables that could be removed without significantly decreasing the explained variance.

## Results

### Number of submissions

A total of 238 parents completed the survey. Data screening identified 33 surveys that had to be excluded because the responding family was a non-Saudi resident living in a different country. The results presented below are based on the final sample of 205 surveys.

### Sample characteristics

Table 1 presents the sample characteristics. Children’s mean age was 7.9 (3.5) years; 26% were < 6 years; 64%, 6–13 years; and 10%, > 14 years. The ratio of affected males to females was 4.9:1, and over 65% of children had additional diagnoses, including attention-deficit/hyperactivity disorder (53%), intellectual disability (8%), epilepsy, and cerebral palsy (2%, each). The average severity of a child's symptoms was 34.9 out of 52 (6.9). The annual household income for 40% of the families was below the sufficiency line (i.e., $28,480), which referred to the amount of income necessary to meet the family's basic needs without public support and included housing, childcare, food, health care, transportation, and entertainment [15].

**Table 1:** Sample characteristics (N = 205)

Variable	N (%)	Median age at diagnosis in years (range)
Overall		3.0 (1.25—6.75)
Child		
Sex		
Male	170 (83)	3.0 (1.25—6.75)
Female	35 (17)	2.8 (1.25—6.00)
Age		
3–5 years	53 (26)	2.3 (1.25—4.75)
6–9 years	109 (53)	3.0 (1.25—5.50)
10–13 years	23 (11)	3.0 (1.25—6.75)
14–18 years	20 (10)	3.3 (1.75—6.00)
Autism red flags		
Language deficit		
Yes	120 (59)	2.9 (1.25—6.75)
No		3.0 (1.25—5.25)
Lack of eye contact		
Yes	100 (49)	2.8 (1.25—6.75)
No		3.0 (1.25—5.25)
Does not respond to name when called		
Yes	115 (56)	2.8 (1.25—6.00)
No		3.0 (1.25—6.75)
Does not smile		
Yes	45 (22)	3.0 (1.25—5.50)
No		3.0 (1.25—6.75)
Lack of looking back and forth to share interests		
Yes	89 (43)	2.7 (1.25—6.75)
No		3.0 (1.25—6.00)
Does not babble		
Yes	25 (12)	3.0 (1.25—5.50)
No		3.0 (1.25—6.75)
Odd play		
Yes	70 (34)	3.0 (1.25—6.75)
No		2.8 (1.25—6.75)
Engaging in challenging behaviors		
Yes	48 (23)	3.0 (1.75—6.75)
No		2.8 (1.25—5.25)
Severity of symptoms		
13–26	21 (10)	2.3 (1.25—4.00)
27–39	133 (65)	3.0 (1.25—6.75)
40–52	51 (25)	3.0 (1.75—6.00)
Comorbidity		
ADHD	109 (54)	3.0 (1.25—6.00)
Intellectual disability	16 (8)	3.5 (2.00—6.75)
Epilepsy	3 (1)	2.3 (2.00—3.50)
Cerebral palsy	3 (1)	3.5 (3.00—4.50)
None	74 (36)	2.5 (1.25—5.25)
City of diagnosis		
Major cities	146 (71)	3.0 (1.25—6.75)
Other cities	41 (20)	2.8 (1.25—5.25)
Outside Saudi Arabia	18 (9)	2.5 (1.50—5.00)
City of diagnosis		
In city of residence	127 (62)	3.0 (1.25—6.75)
Outside city of residence	78 (38)	3.0 (1.25—6.00)
Family		
Saudi citizen		
Yes	184 (90)	2.9 (1.25—6.75)
No	21 (10)	3.0 (1.50—5.25)
Maternal educational attainment		
Less than high school	26 (13)	3.0 (1.25—5.50)
High school degree	47 (23)	3.0 (1.25—6.75)
Some college credits	24 (12)	3.0 (1.25—5.25)
College degree	94 (46)	2.8 (1.25—6.00)
Graduate degree or higher	14 (7)	2.5 (1.75—4.25)
Maternal age		
≤ 24 years	12 (6)	2.6 (1.50–6.75)
25–34 years	100 (49)	3.0 (1.25—5.00)
35–44 years	75 (37)	2.8 (1.25—5.50)
45–54 years	18 (9)	3.0 (1.75—5.50)
Paternal educational attainment		
Less than high school	24 (12)	3.0 (1.25—5.00)
High school degree	50 (24)	3.0 (1.25—6.75)
Some college credits	34 (17)	3.0 (1.25—5.50)
College degree	70 (34)	3.0 (1.25—6.00)
Graduate degree or higher	27 (13)	2.4 (1.75—5.25)
Paternal age		
25–34 years	47 (23)	2.8 (1.25—5.00)
35–44 years	96 (47)	3.0 (1.25—6.75)
45–54 years	45 (22)	2.8 (1.25—6.00)
55–64 years	9 (4)	4.0 (2.00—5.50)
≥ 65 years	7 (3)	3.3 (2.00—5.25)
Annual household income		
Below sufficiency line	81 (40)	3.0 (1.25—5.00)
From sufficiency line to 100% above	81 (40)	3.0 (1.25—6.75)
> 100% above	43 (20)	2.8 (1.25—6.00)
Residence		
Major cities	106 (52)	3.0 (1.25—6.75)
Other cities	94 (46)	3.0 (1.25—6.00)
Outside Saudi Arabia	5 (2)	1.5 (1.25—2.75)

### ASD diagnosis

Most children (71%) were diagnosed in major cities, and over a third (34%), including 55% of those who resided in non-major cities, were diagnosed with ASD outside of their city of residence. Sixty percent of families paid to receive those services. The median age of diagnosis was 3.0 (1.3–6.8) years.

Table 2 presents the unadjusted regression coefficients and associated 95% CIs calculated from the univariate linear models, with the age of diagnosis as the continuous outcome variable. In total, the associations of the age of diagnosis with six demographic and clinical variables were found to be significant at an alpha level = 0.05. These variables included child’s age, the severity of ASD symptoms, lack of response to name, presence of challenging behaviors, lack of comorbidity, comorbid intellectual disability, and residence in a different country. Five variables remained after the backward elimination regression (F (5, 199) = 11.542, *P* < 0.001). With an adjusted R^2^ of 0.2, the model explained 20% of the variance in the age of diagnosis. Table 3 provides the results of the adjusted linear regression predicting the age of ASD diagnosis. A 1 year increase in a child’s age was associated with a 0.1 year increase in age of diagnosis (95% CI 0.05, 0.12). Children who did not respond to their name were diagnosed 0.3 years earlier than other children (95% CI − 0.60, − 0.05), and engaging in challenging behavior was associated with a 0.5 year increase in age of diagnosis (95% CI 0.20, 0.81). A lack of comorbidity was associated with a 0.6 year increase in the age of diagnosis compared to the diagnosis age of children with comorbidity (95% CI 0.13, 1.01). Finally, those residing outside of Saudi Arabia were diagnosed with ASD 0.9 years earlier than those residing in Saudi Arabia (95% CI − 0.171, − 0.11).

**Table 2:** Unadjusted linear regression predicting age of diagnosis in years (N = 205)

Variable	B	SE B	β	95% CI
*Child*				
Sex (male)	− 0.08	0.18	− 0.03	− 0.44 to 0.30
Age (in years)	0.09	0.02	**0.33**	0.06 to 0.13
Citizenship (Saudi)	− 0.26	0.23	− 0.08	− 0.71 to 0.18
Autism red flags				
Language deficit	− 0.04	0.14	− 0.02	− 0.31 to 0.24
Lack of eye contact	− 0.14	0.14	− 0.07	− 0.42 to 0.13
Does not respond to name when called	− 0.31	0.13	− **0.15**	− 0.57 to − 0.29
Does not smile	− 0.11	0.16	− 0.05	− 0.44 to 0.23
Lack of looking back and forth to share interests	− 0.05	0.14	− 0.03	− 0.33 to 0.22
Does not babble	0.03	0.21	0.01	− 0.39 to 0.45
Odd play	− 0.08	0.15	− 0.04	− 0.36 to 0.21
Engaging in challenging behaviors	0.46	0.16	**0.20**	0.15 to 0.78
Severity of symptoms (total score)	0.03	0.01	**0.18**	0.01 to 0.05
Comorbidity				
ADHD	0.12	0.14	0.06	− 0.15 to 0.39
Intellectual disability	0.92	0.26	**0.25**	0.42 to 1.44
Epilepsy	− 0.33	0.58	− 0.04	− 1.46 to 0.81
Cerebral palsy	0.77	0.57	0.09	− 0.36 to 1.90
None	− 0.38	0.15	− **0.18**	− 0.67 to − 0.01
City of diagnosis				
Major cities	0.22	0.15	0.10	− 0.08 to 0.51
Other cities	− 0.15	0.17	0.06	− 0.49 to 0.19
Outside Saudi Arabia	− 0.26	0.24	− 0.08	− 0.74 to 0.22
City of diagnosis (in)	0.01	0.14	0.04	− 0.27 to 0.29
Cost of diagnosis	− 0.1	0.14	0.05	− 0.71 to 0.48
*Family*				
Maternal educational attainment				
Less than high school	0.04	0.21	0.01	− 0.37 to 0.45
High school degree	0.15	0.16	0.06	− 0.18 to 0.47
Some college credits	0.24	0.22	0.07	− 0.19 to 0.66
College degree	− 0.18	0.14	− 0.09	− 0.45 to 0.09
Graduate degree or higher	− 0.19	0.27	− 0.05	− 0.73 to 0.35
Maternal age				
≤ 24 years	0.23	0.3	0.05	− 0.35 to 0.81
25–34 years	− 0.15	0.14	− 0.08	− 0.42 to 0.14
35–44 years	− 0.15	0.14	− 0.73	− 0.39 to 0.18
45–54 years	0.61	0.24	**0.17**	0.13 to 1.08
Paternal educational attainment				
Less than high school	0.01	0.22	0.01	− 0.43 to 0.42
High school degree	0.09	0.16	0.04	− 0.22 to 0.43
Some college credits	0.02	0.19	0.01	− 0.34 to 0.39
College degree	0.09	0.14	0.05	− 0.19 to 0.39
Graduate degree or higher	− 0.4	0.21	− 0.14	− 0.81 to 0.01
Paternal age				
25–34 years	− 0.19	0.16	− 0.08	− 0.51 to 0.14
35–44 years	− 12	0.14	− 0.06	− 0.39 to 0.16
45–54 years	0.05	0.17	0.02	− 0.29 to 0.38
55—65	0.75	0.25	0.20	0.25 to 1.25
Annual household income				
Below the SL	− 0.16	0.14	− 0.08	− 0.44 to 0.12
SL to 100% above	0.25	0.14	0.13	− 0.2 to 0.54
> 100% above	− 0.15	0.017	− 0.06	− 0.48 to 0.19
Residence				
Major cities	0.03	0.14	0.02	− 0.24 to 0.31
Other cities	0.07	0.14	0.03	− 0.20 to 0.34
Outside Saudi Arabia	− 1.03	0.44	− **0.16**	− 1.91 to − 0.16

Statistically significant findings at *P* ≤ 0.05 are in bold

*B* unstandardized beta coefficient, SE* B* standard error, *β* standardized beta coefficient, *CI* confidence interval

**Table 3:** Adjusted linear regression predicting age of diagnosis in years (N = 205)

Variable	B	SE B	β	95% CI
Child age	0.08	0.02	**0.29**	0.05 to 0.12
Not responding to name in the first 2 years of life	− 0.32	0.02	**− 0.16**	− 0.60 to − 0.05
Engaging in challenging behavior in first 2 years of life	0.5	0.16	**0.21**	0.20 to 0.81
No comorbidity	0.61	0.24	**0.16**	0.13 to 1.01
Residence (outside of Saudi Arabia)	− 0.91	0.41	**− 0.14**	− 0.171 to − 0.11

Final model

Statistically significant findings at *P* ≤ 0.05 are in bold

## Discussion

In this study, the age of ASD diagnosis had a low median and was associated with child and family characteristics. This is consistent with the existing literature, which suggests that young children [6, 16, 17], those with moderate to severe symptoms [18, 19], and those with parents with high educational attainment are diagnosed with ASD earlier than their counterparts [7, 17].

Prior studies showed that certain ASD-related behaviors such as toe walking and higher social functioning were associated with earlier diagnosis [20], while greater communication skills and oversensitivity to pain were associated with a later diagnosis [16, 20]. In this study, similarly, not responding to one’s name was associated with an earlier ASD diagnosis, while engaging in challenging behavior was associated with a later ASD diagnosis.

Children of families who resided in other countries were diagnosed earlier than those of families who resided in Saudi Arabia. It is possible that diagnostic services in other countries are better or perhaps provide easier access relative to those in Saudi Arabia. According to a recent study from the United States, the rate of children diagnosed with ASD by 4 years has increased from 58% in 2014 to 71% in 2018 [21].

Comorbid conditions such as major congenital anomalies have been associated with an earlier age at diagnosis [22]. Similarly, our findings indicated that children with no comorbid conditions were diagnosed later than their counterparts. Lack of comorbidity suggests mild ASD symptoms, which are usually associated with a later diagnosis [18].

Greater symptom severity and high socioeconomic status have frequently been associated with an earlier age at diagnosis [7]. In contrast, there was no relationship between these factors and the age at diagnosis in this study. It is possible that we might have had the limited statistical power to identify these potential associations because these characteristics were unbalanced in our small sample. The absence of these relationships could also suggest that diagnostic services were slightly similar across all children, families, and regions in Saudi Arabia.

## Conclusion

This exploratory study was the first to provide information on the age of ASD diagnosis in Saudi Arabia and the factors affecting this age. Although many children were diagnosed early, their parents traveled and paid to receive diagnostic services. More effort needs to be expended to improve these services, especially in non-major cities, to reduce the burden of ASD diagnosis. Establishing public law to ensure free access to early developmental screening with comprehensive diagnostic evaluations and using available technology such as videoconferencing may mitigate the challenges associated with ASD diagnosis [23]. Future research using a large nationally representative sample that examines parents’ experiences and opinions regarding the use of ASD diagnostic services could provide systematic information to improve ASD services and research in Saudi Arabia.

## Limitations

The results of this study should be interpreted in the context of the following limitations. First, the sample was not fully representative of all families of children with ASD in Saudi Arabia, as caregivers who were motivated to complete this survey may differ from non-responders. The sample was likely to exclude families with low educational attainment, families of children with high functioning ASD, and families who did not have access to the Internet. Another limitation was that our survey did not include questions about parents’ satisfaction with the diagnostic services and their interactions with the health and education system. Including such questions would provide a more holistic understanding of the use of ASD diagnostic services as well as explain the differences in age at diagnosis. Nevertheless, the results of this study offer important insight into the factors affecting the age of ASD diagnosis in Saudi Arabia.

## Supplementary Information

**Additional file 1**. A sample of the survey used to conduct this study.

## Data Availability

The datasets used and analysed during the current study are available from the corresponding author on reasonable request.

## Abbreviations

ASD: Autism spectrum disorder
PCQ: Parental concerns questionnaire
CI: Confidence intervals
