# Supplementary material for: Factors associated with age of diagnosis of autism spectrum disorder among children in Saudi Arabia: new insights from a cross-sectional study
Source: BMC Res Notes. 2022 May 10;15:161. doi: 10.1186/s13104-022-06035-x (PMC9092670; doi:10.1186/s13104-022-06035-x)
Supplement: Supplementary file 1 — Additional file 1. A sample of the survey used to conduct this study. [file 13104_2022_6035_MOESM1_ESM.docx]

**Factors Associated with Age of Diagnosis of Autism Spectrum Disorder Among Children in Saudi Arabia: New Insights from A Cross-Sectional Study**

  Fahad Alnemary, *PhD candidate in Department of Human Development and Psychology,* University of California Los Angeles (UCLA), would like to conduct a survey to all parents and/or caregivers of children with ASD in Saudi Arabia to examine *the services received by children with ASD*. Your response to this survey will assist us to identify the *amount and quality of* services provided for children with ASD in Saudi Arabia. The feedback you provide will be shared with policies' makers (Ministry of Health, Ministry of Education, Ministry of Social Affairs).

 Your participation is **extremely important** to us, but it is voluntary and your decision to participate or not participate will not affect the services your child is receiving. You may withdraw from the survey at any time. Note that you are not required to provide identifying information (your or your child's name; or contacts info). This survey will take you 15-20 minutes to complete. (Change after testing)

 **PRIZES**
 To thank you for your time, you will be provided with results of the study.

 **THANK YOU**
 We value your contribution to this effort and thank you for your participation!

 Sincerely,

 Fahad Alnemary, M.A.
 UCLA Graduate School of Education & Information Studies
 Email: falnema2@ucla.edu
 USA cell: 0019517560743
 KSA cell: 0545558618

- Agree to participate

This survey is only for parents or caregivers of children with ASD. Are you a parent (or a caregiver) of a child with autism or autism spectrum disorders?

- YES
- No

How many children with ASD do you have?

- ّI have only one child with ASD
- I have 2 children with ASD
- I have 3 children with ASD
- I have 4 children with ASD
- I have 5 children with ASD
- more (how many?) ________________________________________________

For this survey, you will ***only*** need to provide information about one of your children with ASD.

- click here to provide information about one child with Autism

What is your child's gender?

- Male
- Female

What is your child's DOB? ________________

Citizenship of your child?

- Saudi
- OTHER ________________________________________________

What did you notice during the 1st two years? (Check all apply)

- No or delay in language
- No eye contact
- Not respond to name
- No big smiles
- No back and forth sharing of sounds or smiles
- No babbling
- No gestures such as pointing, showing, reaching, or waving
- Loss of speech, babbling or social skills
- Engaging in challenging behavior
- Not plying with toy functional (e.g., line up black, spinning car’s wheel, etc.)
- Other ________________________________________________

At what age child was the child diagnosed?

| Year | Month |
| --- | --- |
|  |  |
| ____________________ | ____________________ |

At which city was your child diagnosed? ____________________

You indicated that your child was diagnosed out of KSA, please indicate the country and the city at where you child was diagnosed? ____________________

Some children with ASD might have other conditions or mental disorders. Is there any other condition or mental disorder the child diagnosed with? Choose one of the following:   (Check all that applies)

- No
- Cerebral palsy
- Epilepsy
- Attention-deficit/hyperactivity disorders
- Intellectual disability
- Other ________________________________________________

What is the mother current age?

- Less than 16
- 16 to 19
- 20 to 24
- 25 to 34
- 35 to 44
- 45 to 54
- 55 to 64
- 65 or over

What is father current age?

- Less than 16
- 16 to 19
- 20 to 24
- 25 to 34
- 35 to 44
- 45 to 54
- 55 to 64
- 65 or over

**What is the highest educational degree the mother earned?**

- No schooling completed
- No schooling completed
- No schooling completed
- High school
- Some college credits
- BA
- Graduate and/or higher

**What is the highest educational degree the father earned?**

- No schooling completed
- Elementary school
- Middle school
- High school
- Some college credits
- BA
- Graduate and/or higher

**Mother's Employment Status**

- Employed for wages
- Self-employed
- Out of work and looking for work
- Out of work but not currently looking for work
- A homemaker
- A student
- Retired
- Unable to work
- Other ________________________________________________

**Father's Employment Status**

- Employed for wages
- Self-employed
- Out of work and looking for work
- Out of work but not currently looking for work
- A homemaker
- A student
- Retired
- Unable to work
- Other ________________________________________________

What is your combined annual household income?

- Less than 5,000
- 5,000 – 9,999
- 10,000 – 14,999
- 15,000 – 19,999
- 20,000 – 24,999
- 25,000 – 29,999
- 30,000 – 34,999
- 35,000 – 39,999
- 40,000 or more

At which city does the child live? ________________

You indicated that your child lives out of KSA, please indicate the country and the city at where does your child live? ____________________

For each of the following statements, please check the one option that best describes the child.

|  | Strongly disagree (1) | Disagree (2) | Agree (3) | Strongly Agree (4) | not clear question (5) |
| --- | --- | --- | --- | --- | --- |
| My child doesn’t use words, has difficulty initiating conversations. (1) |  |  |  |  |  |
| My child completes routines always in the same manner. (2) |  |  |  |  |  |
| My child shows distress from new situations or crowds. (3) |  |  |  |  |  |
| My child has sensory issues (e.g., reacts differently to lights, sounds, textures). (4) |  |  |  |  |  |
| My child has sleeps issues (e.g. he does not fall asleep easily and/or wakes often). (5) |  |  |  |  |  |
| My child intentionally hits and/or bites others. (6) |  |  |  |  |  |
| My child is constantly moving, jumping, and/or running (7) |  |  |  |  |  |
| My child has difficulty finishing a task. (8) |  |  |  |  |  |
| My child has unpredictable changes between emotions. (9) |  |  |  |  |  |
| My child eats few foods/certain types of food. (10) |  |  |  |  |  |
| My child prefers to be alone, has few friends. (11) |  |  |  |  |  |
| My child rocks, spins, and/or flap hands. (12) |  |  |  |  |  |
| My child bangs head, pinches, bites, and/or hits him/herself. (13) |  |  |  |  |  |
